# 2021 Editor’s Choice Articles in the Section “Cells of the Nervous System”

**DOI:** 10.3390/cells11233802

**Published:** 2022-11-28

**Authors:** Naweed I. Syed

**Affiliations:** Hotchkiss Brain Institute, Cumming School of Medicine, University of Calgary, Calgary, AB T2N 4N1, Canada; nisyed@ucalgary.ca

Referenced below are the top 10 cited papers in 2021 from the Section “Cells of the Nervous System”, published in *Cells* (ISSN: 2073-4409). The rationale behind selecting these papers was that they all make significant advances in the field of Neurosciences—with specific emphasis on various neurodegenerative diseases and the identification of their potential mechanisms. Several studies among them offer future therapeutic potential for various compounds that had not previously been investigated extensively. The selected papers are topical, timely and contribute significantly to the field of Neurosciences. 

All animal functions—ranging from simple reflexes to learning, memory and higher cognitive functions—rely upon several tens of billions of neurons, which are interconnected through specialized structures, termed the synapses. Perturbation of neuronal connectivity or communications resulting from developmental disorders, injury, trauma, stroke, neuro-inflammation, neurodegenerative diseases—such as Alzheimer’s (AD), Parkinson’s, Amyotrophic Lateral Sclerosis (ALS)—etc., compromise myriad brain functions. Unfortunately, our understanding of various mechanisms underlying brain ailments remains limited—restricting therapeutic potential to symptom management alone. The selected papers highlighted here provide insights into the role of various cell types in normal brain functions, as well as their roles when degenerative processes begin to take hold. 

It was initially thought that the synaptic transmission between neurons alone generated the patterns of connectivity underlying all brain functions. Glial cells, which although outnumbered neurons, were deemed to merely serve the function of a glue holding the neuronal architecture together. In some other instances, they were deemed to be either the providers of nourishment for neurons or as repair workers after brain injury. To our surprise, we have since learned that the glial cells do more for our brain functionality than the roles to which they had been previously ascribed. It is now well established that in addition to serving several housekeeping functions, the glia (astrocytes, oligodendrocytes, etc.) exhibit a tri-partied relationship with neuronal cells whereby they serve either to augment or curtail the intensity, frequency and efficacy of neuronal communications. Furthermore, whereas our initial assumptions were that the glial cells were perhaps only engaged during normal synaptic transmission, they have now been shown to play important roles in both the exacerbation of diseases such as AD or ALS and in coming to the rescue of a degenerating brain [1,2]. These two papers not only highlight the pathological involvement of astrocytes in AD but also search for underlying mechanisms involved. The study by Raffaele et al. [2] examined the role of oligodendrocytes in ALS and alluded towards several pathways that can be invoked to seek remedy. Scaricamazza et al. [3], on the other hand, suggest a paradigm shift in the treatment of ALS by focusing primarily on the skeletal muscles rather than various neuronal cell types. As all current interventions to subdue plagues and tangles caused by beta amyloid and tau proteins being the causative agents of AD have failed, Chen et al. [4] offer several natural products as a potential remedy. Although the clinical potential of these compounds remains to be fully examined, the data presented do, however, suggest that it is now time to look past the adhesion plaque models and examine alternative causes, causative agents and remedies for AD. Similarly, Tribble et al. [5] demonstrate how a specific diet and exercise regime could serve as a neuroprotective agent and recovery mechanism in animal models of glaucoma. Along the same line, Mithaiwala et al. [6] implicate neuroinflammation and Kynurenine pathways in several central nervous system (CNS) diseases as they seek to deduce its therapeutic potential and that of the underlying cellular and molecular pathways involved.

Like AD, Parkinson's disease is a debilitating neurodegenerative disorder, affecting millions around the world; its symptoms, at best, can only be managed through drugs [7], with there being no cure in sight. Whereas many drugs prescribed to Parkinsonian patients may ameliorate motor dysfunction caused by dopamine deficiency, in so doing, they also disrupt the balance between aminergic systems, giving rise to mood disorders, etc. Vallee et al. [7] set out to test the potential actions of lithium targeting the WNT/B- catenin pathway, oxidative stress, neuroinflammation and the glutamatergic pathway. Although the potential clinical significance of this “short-gun” approach and the precise interdependence of myriad targeted pathways remains elusive, these studies nevertheless underscore the potential of further exploring lithium as a putative therapeutic agent. 

Spernaza et al. [8] examine in detail the role of dopamine from the view point of a classical transmitter and its involvement in synaptic plasticity and the control of motor movement. This and other similar studies serve to demonstrate that ascribing any specific function to a given neurotransmitter may be naïve and that its potential involvement vis-à-vis different brain functions must be investigated independently. Why should this be important? It is important in the context of various drug therapies or other non-pharmacological treatment regimens that, although they alter brain function in one region, it ought not to be at the expense of changing neuronal functionality in other areas. Specifically, one must ensure that the perturbation of one neuronal pathway does not affect the other brain regions where the same neurotransmitter might be serving a different function. Along the same lines, Gardoni et al. [9] show an alternative to drug therapy where they targeted NMDA and AMPA receptor autoantibodies to not only decipher the potential pathways involved but also to demonstrate their therapeutic potential for brain ailments in which these transmitters and their receptors may be involved. 

Finally, we selected an article published by Shukla et al., [10] as it highlights the interplay between the gut–microbial–inflammasome–brain axis in a mouse model of AD. This gut–brain connection has recently been found to play an important role in the regulation of several brain functions, and this study provides some interesting and novel insights into how microbial flora and the emanating metabolites may affect not only the function of sympathetic and parasympathetic nervous systems but also the CNS. 

I anticipate that the follow-up studies from these selected papers will open novel and interesting avenues of research, and I look forward to further progress—both at the mechanistic level and also in their therapeutic potential.
